# Composition of the Midgut Microbiota Structure of *Haemaphysalis longicornis* Tick Parasitizing Tiger and Deer

**DOI:** 10.3390/ani14111557

**Published:** 2024-05-24

**Authors:** Zi-Ling Liu, Qi-Guan Qiu, Tian-Yin Cheng, Guo-Hua Liu, Lei Liu, De-Yong Duan

**Affiliations:** 1 Research Center for Parasites & Vectors, College of Veterinary Medicine, Hunan Agricultural University, Changsha 410128, China; zl339569@126.com (Z.-L.L.); hn5368@163.com (T.-Y.C.); liuguohua5202008@163.com (G.-H.L.); 2 Changsha Ecological Zoo, Changsha 410128, China; 48191372@163.com

**Keywords:** *Haemaphysalis longicornis*, microbiome, amplicon sequencing, vector-host interactions

## Abstract

**Simple Summary:**

*Haemaphysalis longicornis* is a predominant tick species in East Asia and infests humans, cattle and wildlife. It is also a common vector for pathogen transmission. The microbial community in tick midguts not only affects the transmission of tick-borne pathogens but also indirectly affects the development, metabolism, reproduction, and other physiology of ticks. There have been rare reports on the effects of different types of hosts on the microbial composition of tick midguts. Here, we conducted a midgut microbiome of *H. longicornis* collected from tiger and deer by high-throughput sequencing and showed that the relative abundance of each bacterium varied greatly at different classification levels.

**Abstract:**

*Haemaphysalis longicornis* is a common tick species that carries several pathogens. There are few reports on the influence of different hosts on the structure of midgut microflora in *H. longicornis*. In this study, midgut contents of fully engorged female *H. longicornis* were collected from the surface of tiger (*Panthera tigris*) and deer (*Dama dama*). The bacterial genomic DNA of each sample was extracted, and the V3–V4 regions of the bacterial 16S rRNA were sequenced using the Illumina NovaSeq sequencing. The diversity of the bacterial community of the fully engorged female *H. longicornis* on the surface of tiger was higher than that of deer. In total, 8 phyla and 73 genera of bacteria annotations were detected in the two groups. At the phylum level, the bacterial phyla common to the two groups were Proteobacteria, Firmicutes, and Actinobacteriota. At the genus level, there were 20 common bacterial genera, among which the relative abundances of *Coxiella*, *Morganella*, *Diplorickettsia*, and *Acinetobacter* were high. The *Morganella* species was further identified to be *Morganella morganii*. The alpha diversity index indicated that the bacterial diversity of the tiger group was higher than that of the deer group. Bacteroidota, Patescibacteria, Desulfobacterota, Verrucomicrobiota, and Cyanobacteria were solely detected in the tiger group. A total of 52 bacterial genera were unique in the tiger group, while one bacterial genus was unique in the deer group. This study indicates that there are differences in the structure of the gut bacteria of the same tick species among different hosts. Further culture-based methods are needed to provide a more comprehensive understanding of the tick microbiota parasitizing different hosts.

## 1. Introduction

The *Haemaphysalis longicornis* tick belongs to the genus *Haemaphysalis* of the ixodes family. *H. longicornis* is distributed in the United States, Korea, Japan, Russia, New Zealand, Australia, and other countries [1,2]. It is also a common tick species in northeastern and central China [2]. *H. longicornis* has a wide range of hosts; these hosts mainly are deer, cattle, sheep, brown rabbits, and dogs [3]. The direct harms of these ticks to hosts include paralysis, anemia, emaciation, and decreased fur quality [4]. More importantly, they act as vectors for several pathogens that can be transmitted to humans and animals during the blood-sucking process [5]. In addition, some pathogens, including tick-borne encephalitis virus (TBEV) [6], severe fever with thrombocytopenia syndrome virus (SFTSV) [7], *Anaplasma* [8], *Rickettsia*, *Borrelia burgdorferi*, and other pathogens [9], have been detected in *H. longicornis*. Thus, pathogen species and their abundance with in ticks need a concerned investigation for ensuring the public health.

As the largest organ in ticks, the midgut is the key interface for pathogen survival, colonization, and infection [10,11]. Simultaneously, symbiotic bacteria in ticks are active in midgut tissues and can provide nutrients for the growth and development of ticks [12]. The pathogens, symbiotic bacteria, and others together constitute the midgut microbiota of ticks. The diversity of the tick midgut microbiome may be affected by the geographical environment, host, temperature, developmental stage, sex, and other factors [13]. The structure of *H. longicornis* microflora has been reported in previous studies. Proteobacteria was identified to be the dominant phylum among *H. longicornis* [14]. *Coxiella* is the most abundant bacterial genus, and its relative abundance gradually increases with the blood-sucking process of *H. longicornis* at different developmental stages [14]. These results provide a reliable basis for the recognition of midgut pathogens and symbiotic species of *H. longicornis*. The microbiome from *H. longicornis* ticks is affected by different habitats, hosts, sampling sites, and other factors [15]. However, the influence of different hosts on the midgut microflora structure of *H. longicornis* has not studied extensively.

In this study, *H. longicornis* ticks from different hosts were collected, and their midgut microflora structure was analyzed using the Illumina NovaSeq sequencing platform. The resulting double-terminal reads were spliced and filtered to obtain amplicon sequence variants (ASVs), followed by species annotation, abundance, and alpha diversity analyses to reveal the midgut microbiome of *H. longicornis* parasitizing tiger and deer. Therefore, the purpose of this study was to investigate whether there were differences in the structure of the gut bacteria of the same tick species among different hosts.

## 2. Materials and Methods

### 2.1. Collection and Identification of Tick Samples

**Figure 1:**
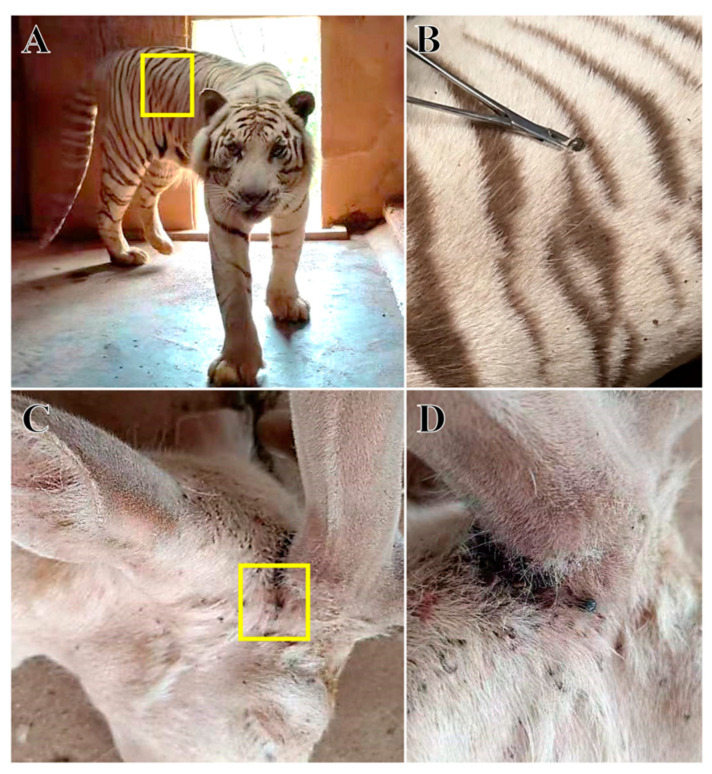
Sample collection. (**A**), The tiger host; (**B**), further details showing ticks on the surface of tiger; (**C**), the deer host; (**D**) further details showing ticks on the surface of deer.

Fifty female ticks with different satiated blood statuses were collected from the surface of tiger (Figure 1A,B) and deer (Figure 1C,D) at Changsha Ecological Zoo (28°1′ N, 113°0′ E), Hunan Province, China. The sample size referred to a recent similar study [16]. The tigers and deer lived in a stable environment within the same zoo, but two types of animals were housed in a relatively distant area (>0.5 km). Tigers were free-ranging in an outdoor enclosure with trees and shrubs as the main vegetation. In contrast, deer were kept in a smaller enclosed area with a cement-hardened ground and a few grasses. The zookeepers reported the tick infection during the weekly routine inspection. All tick samples were collected from naturally infected hosts at the same time.

A total of 5 mL of host blood was collected using disposable intravenous needles by practicing veterinarians. The blood sample of tiger was collected from the caudal vein, and the blood sample of deer was collected from the jugular vein. Tick samples and host blood samples were immediately sent to the Research Center for Parasites & Vectors, Hunan Agricultural University for further analysis.

### 2.2. Identification of Tick Species

The ticks were observed under a stereomicroscope (Nikon, Tokyo, Japan). According to the morphological description by Teng and Jiang (1991), tick species collected in this study were preliminarily identified to be *H. longicornis* [17].

According to previous reports [18,19], amplification primers for the tick 16S rDNA and COX1 genes and ITS sequences were designed and synthesized by Sangon Biotech (Shanghai, China). The sequences of primers used are listed in Table 1. Tick DNA was extracted using a genomic DNA extraction kit (TianGen Biotech, Beijing, China). The PCR amplification system was as follows: DNA template1 μL, upper and downstream primers (10 mM) 1 μL, 2 × Easy TaqPCR Super Mix (Accurate Biotech, Changsha, China) 25 μL, and ddH_2_O 22 μL. The amplification conditions for the COX1 gene were as follows: initial denaturation at 95 °C for 5 min, followed by 40 cycles of 95 °C for 30 s, 55 °C for 60 s, 72 °C for 60 s, and a final elongation at 72 °C for 5 min. The amplification conditions for the ITS sequences were as follows: initial denaturation at 95 °C for 5 min, followed by 35 cycles of 95 °C for 30 s, 54 °C for 90 s, and 72 °C for 60 s, and a final elongation at 72 °C for 5 min. The amplification conditions for the 16S rDNA gene were as follows: initial denaturation at 95 °C for 5 min, followed by 35 cycles at 95 °C for 30 s, 56 °C for 30 s, and 72 °C for 60 s, and a final elongation at 72 °C for 5 min. The amplification products (4 μL) were subjected to electrophoresis on a 15 g/L agarose gel. The target bands were cut, and the PCR products were recovered using a DNA rapid recovery and purification kit (TianGen Biotech). The recovered products were connected to the pEASY-T1 cloning vector (TransGen Biotech, Beijing, China), and positive clones were sequenced by Sangon Biotech (Shanghai, China). The sequences obtained were subjected to multiple sequence analysis to identify tick species. MEGA7 software (v7.0.26) was used to construct a phylogenetic tree of each gene via the neighbor-joining (NJ) and maximum-likelihood (ML) methods.

### 2.3. DNA Extraction of the Midgut Microbiota and Blood Extraction

Five fully engorged ticks (0.280 ± 0.005 g) were used for each group. The ticks were wiped and disinfected with 75% alcohol and washed with sterile water three times to remove the impurities on body surface. The last wash water was used as the negative control. The marginal groove of the ticks was cut off, and the dorsum of the ticks was removed to avoid the outflow of midgut content. Then, the ticks and their organs were suspended in saline solution and exposed under a stereomicroscope. The other organs were separated from the midguts by a needle. The midgut tissues and contents were transferred to sterilized centrifuge tubes containing 1 mL of 3.8% sodium citrate sterile saline solution. The supernatant was centrifuged at 8609× *g* for 1 min. The supernatant was discarded, and the deposits were retained. The microbial genomic DNA of the deposits and the host whole blood samples were extracted using a Genomic DNA Extraction Kit (TianGen Biotech).

### 2.4. 16S rRNA Gene Amplification and High-Throughput Sequencing

The V3-V4 regions of bacterial 16S rRNA were amplified using primers 341F (5′-CCT AYG GGR BGC ASC AG-3′) and 806R (5′-GGA CTA CNN GGG TAT CTA AT-3′) with different barcodes. The bacterial genomic DNA of the two groups, the host blood genomic DNA, and the last wash water were used as templates for PCR amplification. The PCR amplification system was as follows (50 μL in total): DNA template 1 μL, Phusion^®^ High-Fidelity PCR Master Mix (New England Biolabs, Ipswich, MA, USA) 25 μL, upper and downstream primers (10 mM) 2 μL, and ddH_2_O 20 μL. The amplification conditions were as follows: 98 °C for 1 min; 98 °C for 10 s, 50 °C for 30 s, and 72 °C for 30 s for a total of 30 cycles; and 72 °C for 5 min. The PCR products were detected by 2% agarose gel electrophoresis. In parallel with each amplification, a negative control (distilled water) was included. Electrophoresis of the host blood genomic DNA and the wash water revealed negative results. The results of the bacterial genomic DNA of the two host groups were positive. Because the host blood genomic DNA groups and wash water groups were unavailable for library construction, they were excluded from further study. Then, 16S rRNA gene amplicons from the two groups were recovered using a rapid DNA fragment purification and recovery kit (Qiagen, Düsseldorf, Germany). The NEBNext^®^ Ultra™ II DNA Library Prep Kit (New England Biolabs) was used for library construction. Each amplicon was amplified three times. After the libraries were quantified by a Qubit@ 2.0 fluorometer (Thermo Scientific, Waltham, MA, USA) and quantitative real-time polymerase chain reaction, sequencing was performed using the Illumina NovaSeq platform (Thermo Fisher, Waltham, MA, USA).

### 2.5. Data Analysis

#### 2.5.1. Sequencing Data Processing

After the barcode and primer sequences were removed, reads were spliced using FLASH software (version 1.2.11, http://ccb.jhu.edu/software/FLASH/, accessed on 1 August 2022) to obtain the raw tags. Then, fastp software (https://github.com/OpenGene/fastp, accessed on 1 August 2022) was used to control the raw tags, and high-quality clean tags were obtained. Finally, Vsearch software (https://github.com/torognes/vsearch, accessed on 1 August 2022) was used to compare with the database to detect and remove chimeras to obtain the final data, that is, the effective tags.

#### 2.5.2. Amplicon Sequence Variant Noise Reduction and Species Annotation

For the effective tags, the DADA2 [20] module in QIIME2 software (https://qiime2.org/, accessed on 1 August 2022) was used to filter out sequences with an abundance of less than 5 to obtain the final Amplicon sequence variants ASVs corresponding to OTUs (which represent sequences) and the feature list. Then, the classify-sklearn [21,22] module in QIIME2 software was used to identify the ASVs [23]. The Silva138.1 database was used to annotate species information for each of the amplicon sequence variants (ASVs) [24]. The community composition of the two groups was determined at different levels of classification.

#### 2.5.3. Alpha Diversity Analysis

QIIME2 software was used to calculate the observed ASVs, and Shannon, Simpson, Chao1, and Good’s coverage indices were employed to analyze the intergroup differences in alpha diversity. The rarefaction curves were displayed using Image GP (Version 1.0) software following these indices.

#### 2.5.4. Beta Diversity Analysis

Based on phylogenetic group ASVs, the unweighted unifrac distance, Jaccard distance and Bray Curtis distance were calculated to measure the coefficient of difference between two groups through QIME2 platform.

#### 2.5.5. Identification of *Morganella* in Ticks

According to a previous study [25], the amplification primers for the 16S rDNA, *dnaN*, *tuf*, and *hdc* genes of *Morganella* were designed and synthesized by Sangon Biotech (Shanghai, China). The sequences of primers used are listed in Table 1. The 16S rDNA, *dnaN*, *tuf*, and *hdc* genes were amplified using PCR reaction as follows: total DNA template of tick midgut bacteria 1 μL, primers for the upper and downstream regions 1 μL (10 mM), 2 × EasyTaq PCR Super Mix (Accurate Biotech) 25 μL, ddH_2_O 22 μL. In parallel, a negative control (distilled water as a template) was included.

The amplification conditions for the 16S rDNA gene were as follows: initial denaturation at 95 °C for 30 s, followed by 34 cycles of 95 °C for 20 s, 51 °C for 20 s, and 72 °C for 60 s, and a final elongation at 72 °C for 5 min. The amplification conditions for the *dnaN* gene were as follows: initial denaturation at 95 °C for 30 s, followed by 34 cycles of 95 °C for 20 s, 51 °C for 20 s, and 72 °C for 60 s, and a final elongation at 72 °C for 5 min. The amplification conditions for the *tuf* gene were as follows: initial denaturation at 95 °C for 30 s, followed by 34 cycles of 95 °C for 20 s, 52 °C for 20 s, 72 °C for 60 s, and a final elongation at 72 °C for 5 min. The amplification conditions for the *hdc* gene were as follows: initial denaturation at 95 °C for 30 s, followed by 34 cycles of 95 °C for 20 s, 55 °C for 20 s, and 72 °C for 60 s, and a final elongation at 72 °C for 5 min. Amplified products of 5 μL were obtained and subjected to electrophoresis on a 15 g/L agarose gel. The target bands were cut, and the PCR products were recovered using a DNA rapid recovery and purification kit. The recovered products were connected to the pEASY-T1 cloning vector (TransGen Biotech, Beijing, China), and the positive clones were sequenced by Sangon Biotech. The sequences obtained were compared and analyzed with existing sequences in GenBank. Based on the homology of each gene sequences, MEGA7 software (v7.0.26) was used to construct a phylogenetic tree of each gene via the NJ and ML methods.

## 3. Results

### 3.1. Identification of Ticks

**Figure 2:**
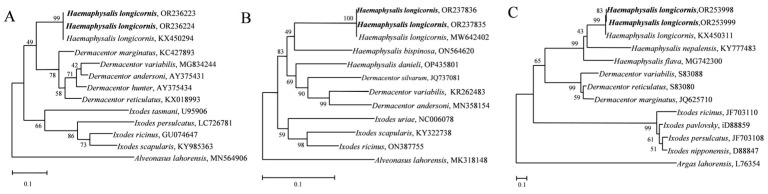
Phylogenetic tree based on 16S rDNA, COX1 and ITS sequences. (**A**), 16S rDNA; (**B**), COX1; (**C**), ITS. Phylogenetic trees were constructed based on neighbor-joining (NJ; 1000 bootstrap replicates) and maximum-likelihood (ML; 1000 bootstrap replicates) analyses using MEGA7. The scale bar represents the inferred substitutions per nucleotide site. The relative support for clades in the tree was produced from the NJ and ML analyses.

The ticks in the tiger and deer groups were morphologically identified as *H. longicornis*. Further molecular identification of the two groups were performed using 16S rDNA and COX1 genes and ITS sequences, in which the 16S rDNA gene was successfully amplified at 347 bp (tiger) and 372 bp (deer). COX1 was successfully amplified at 777 bp (tiger) and 775 bp (deer), and the ITS sequences were also successfully amplified at 1773 bp (tiger) and 1802 bp (deer) (Figure S1). The obtained gene sequences were uploaded to the NCBI database with the following accession numbers for 16S rDNA: OR236224 and OR236223; COX1: OR237835 and OR237836; and ITS: OR253998 and OR253999. Phylogenetic trees were constructed using the above gene sequences. All phylogenetic trees had a well aggregation, thus supporting the identification of ticks to be *H. longicornis* in this study (Figure 2). Morphological and molecular biology analyses showed that the ticks on the surface of tigers (Figure 1A,B) and deer (Figure 1C,D) in the Changsha Ecological Zoo in Hunan Province were the *H. longicornis* ticks.

### 3.2. Data Processing and ASVs Statistical Results

In the original sequence obtained by sequencing, the two groups generated 147,802 raw reads on the Illumina NovaSeq sequencing platform. After removing the barcode and primer sequences, 46,597 and 71,331 effective sequences were obtained from tiger and deer groups for next analyses, respectively. After noise reduction, 138 ASVs were obtained for the tiger group and 28 ASVs were obtained for the deer group, among which 22 ASVs were shared by the two groups, and six ASVs were unique to the deer group.

### 3.3. Analysis of Microbial Compositions

**Figure 3:**
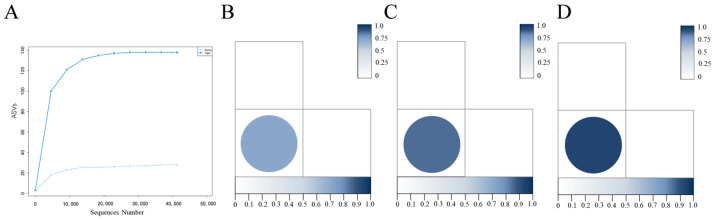
Rarefaction curve, unweighted unifrac distance, Jaccard distance, Bray Curtis distance of midgut samples from fully engorged female adult *H. longicornis* parasitizing tiger and deer. (**A**) Rarefaction curves; (**B**–**D**) unweighted unifrac distance (dissimilarity value = 0.82), Jaccard distance (dissimilarity value = 0.84), Bray Curtis distance (dissimilarity value = 0.94).

The alpha diversity indices of the tiger and deer groups were analyzed (Table 2). The results show that the Goods coverage of tiger and deer were 1.000, indicating that the sequencing depth in two groups were sufficient, and the data volume could be used for the corresponding analysis. The Chao1, Shannon, and Simpson indices of the tiger group were higher than those of the deer group, indicating that there were a large number of low-abundance species in the midgut community of *H. longicornis* parasitizing tigers. The rarefaction curve (Figure 3A) showed that the bacterial diversity of tiger group was higher than that of deer group. Unweighted unifrac distance (Figure 3B), Jaccard distance (Figure 3C), and Bray curtis distance (Figure 3D) showed that there were significant differences in the bacterial diversity between the tiger and deer group.

### 3.4. Difference Analysis of Midgut Microbiota Structure

**Figure 4:**
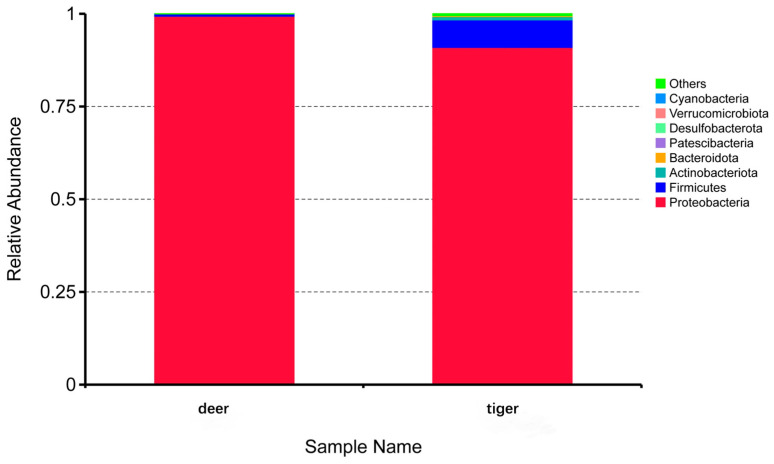
Relative abundances of bacteria at the phylum level. ‘Others’ indicates the sum of the relative abundances of the phyla except for the top 8 ones.

After species annotation of ASVs, eight phyla were found in two groups and the relative abundance of each phylum is shown in Figure 4. There were three common phyla in the two groups, among which Proteobacteria had the highest relative abundance, accounting for 99.4% (deer) and 90.9% (tiger) of the total phyla. In addition, Firmicutes and Actinobacteriota were also commensal phyla taxa in two groups. In addition to common bacteria, Bacteroidota, Patescibacteria, Desulfobacterota, Verrucomicrobiota, and Cyanobacteria were unique bacteria in the tiger group.

**Figure 5:**
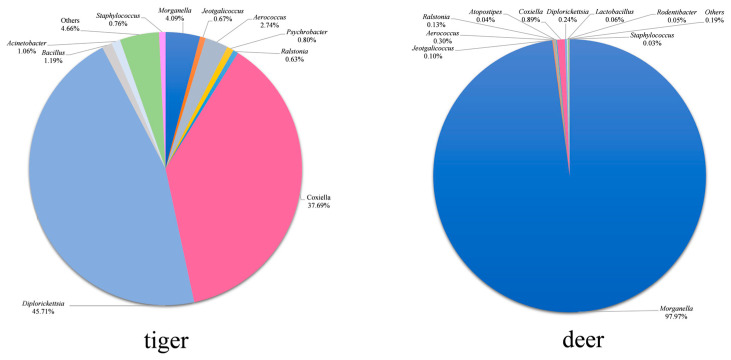
Relative abundances of bacteria at the genus level (top 10). ‘Others’ indicates the sum of the relative abundances of the genera except for the top 10 ones.

A total of 73 bacterial genera were detected in *H. longicornis* from both groups listed in Table S1. The distribution characteristics of the top 10 common bacterial genera with relative abundances in the samples of the two groups are shown in Figure 5, and the relative abundances of other common bacterial genera are shown in Table S1. *Diplorickettsia*, *Coxiella*, *Morganella*, *Aerococcus*, *Bacillus*, and *Acinetobacter* were the high-abundance bacterial genera (above 1%) in the tiger group; *Morganella*, *Coxiella*, *Aerococcus*, *Diplorickettsia*, *Ralstonia*, and Jeotgalicoccus were the high-abundance ones in the deer group; *Diplorickettsia*, *Coxiella*, *Morganella*, *Aerococcus* were high-abundance ones in both groups. *Diplorickettsia* was the highest in the tiger group, accounting for 45.7%, while *Morganella* was the highest in the deer group, accounting for 97.9%. A total of 52 bacterial genera were unique in the tiger group, and one bacterial genus was unique in the deer group.

### 3.5. Identification of Morganella 

**Figure 6:**
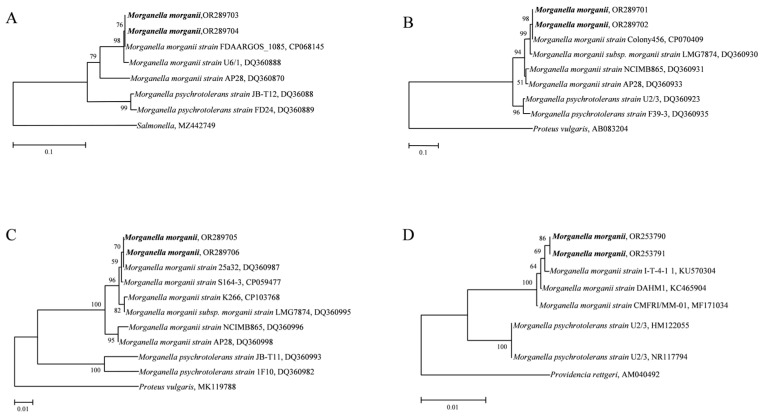
Phylogenetic trees based on *dnaN*, *hdc*, *tuf* and 16S rDNA sequences of *Morganella*. (**A**). *dnaN*. (**B**). *hdc*. (**C**). *tuf*. (**D**). 16S rDNA. The phylogenetic trees were constructed on the basis of neighbor-joining (NJ; 1000 bootstrap replicates) and maximum-likelihood (ML; 1000 bootstrap replicates) analyses using MEGA7. The scale bar represents the inferred substitutions per nucleotide site. The relative support for clades in the tree was produced from the NJ and ML analyses.

The 16S rDNA, *dnaN*, *tuf*, and *hdc* genes were used to identify the *Morganella* specie in the two groups. Phylogenetic trees of the four genes showed that the amplified *Morganella* genes in both the deer and tiger groups polymerized with *M. morganii* to form a branch, and *M. psychrotolerans* formed a branch alone. All four phylogenetic trees were topologically stable. The high confidence indicates that the genus *Morganella* in the two groups was *M. morganii* (Figure 6). All sequences were uploaded to the GenBank database (16S rDNA: OR253790, OR253791; *dnaN*: OR289703, OR289704; *tuf*: OR289705, OR289706; *hdc*: OR289701, OR289702.)

## 4. Discussion

Ticks can acquire different bacteria from the host, reproductive process, and environment throughout their life cycle, forming a relatively stable microflora structure in the midgut [26]. The tick microbiome includes disease-causing symbiotes that can influence tick biology and vector capacity to regulate pathogen infection, reproduction, and spreading [27]. The diversity of a tick midgut microbial community is influenced by its host [13]. 

Therefore, we performed a high-throughput sequencing targeting distinction of the microbial community of *H. longicornis*, which were collected from tiger and deer. There were 8 and 73 bacterial taxa in the two groups at the phylum and genus levels, respectively. The bacterial taxa shared by the two groups were 3 and 20 at the phylum and genus levels, respectively. Besides the commensal taxa, 5 phyla and 52 genera were unique in the tiger group. The alpha diversity index indicates that midgut community diversity from the tiger group was higher than that from the deer group. The gut microbiome structure in the tiger and deer groups varied greatly in terms of the relative abundance of each bacterium at different taxonomic levels. The tiger group showed a large number of low-abundance species in the midgut community and higher bacterial diversity. This variation may be due to differences in the host genotype, health status, or host environment [15].

Ticks can carry obligate and facultative symbionts that are passed from the mother to the offspring [28]. Both types of symbionts are associated with the health of ticks, and the elimination of symbionts has adverse effects on laying, egg hatching, and survival of ticks [29]. In addition, these symbionts also affect the acquisition, colonization, and spread of tick-borne pathogens [30,31].

*Coxiella* was detected in the midgut of *H. longicornis* ticks. Currently, *Coxiella burnetii* and *Coxiella* endosymbionts have been found in ticks. *C. burnetii* is a lethal bacterium that causes Q fever and is considered a medically significant zoonotic pathogen worldwide [32]. This pathogen was first isolated from hard ticks *Dermacentor andersoni* and *H. humerosa* [33]. *C. burnetii* has been found in over 40 species of ticks, including *Rhipicephalus* spp. and *Dermacentor* spp., suggesting that these tick species are vectors for the transmission of Q fever [34]. *Coxiella* endosymbiont is common in tick species, including *Amblyomma americanum* [35], *Ornithodoros rostratus* [36], *Rhipicephalus turanicus*, *R. sanguineus* [37], *Ornithodoros* (*Carios*) *capensis* (Argasidae) [38] and *H. longicornis* [39]. Ticks can feed exclusively on vertebrate blood because of the ability of intracellular bacterial symbionts to synthesize B vitamins [40,41]. These nutritional endosymbionts are essential to the life cycle of ticks. In the case of the *Coxiella*-like endosymbiont of *H. longicornis*, metagenomics has shown that *Coxiella*-like endosymbionts can produce the three key B vitamins folic acid, biotin, and riboflavin required for tick well-being. *Coxiella*-like endosymbiont also plays an important role in determining the reproductive adaptability of ticks [42].

*Acinetobacter* is an aerobic saprophyte with an oxidation metabolism that is widely distributed in water, soil, and other external environments. Currently, 53 *Acinetobacter* species have been identified and classified, including opportunistic pathogens and potentially pathogenic strains [43]. *Acinetobacter* in *Ixodes holocyclus*, *Rhipicephalus* (*Boophilus*) *decoloratus*, *Acinetobacter triguttatum*, *Aponomma fimbriatum*, *Bovicola* spp. (ex-goat), and *Bovicola* spp. (ex-alpaca) have been extensively studied in blood-sucking arthropods [44]. In a study by Clayton et al. (2015), *Acinetobacter* was found to be one of the major symbionts in the midgut and salivary glands of *Dermacentor andersoni*. The amount of *Acinetobacter* in ticks may depend on the length of time that it was exposed to the surface of the host [45]. In this study, *Acinetobacter* was detected in both the deer and tiger groups, and its relative abundance in tiger group was higher than that in deer group. These results indicate that *Acinetobacter* can stably colonize the midgut of *H. longicornis* and that its abundance can change among different hosts.

*Staphylococcus* includes at least 66 effective species, including coagulase-negative *Staphylococcus* (CoNS), *S. lentus*, *S. saprophyticus*, *S. aureus*, *S. epidermidis* and others [46]. Huang et al. (2019) reported that *Staphylococcus* had a high abundance of 8.04–98.76% among ticks in the Yuyao area of China [47]. *Staphylococcus* is often found in *A. americanum* and *R. microplus* and may be derived from the skin and fur of the host [48,49]. It is common for ticks to carry *Staphylococcus* from those reports. Our results show that *Staphylococcus* was present in the tiger and deer groups, and relative abundance of *Staphylococcus* in the tiger samples was higher than that in the deer sample. *S. lentus* was the reported cause of endometrial infection in European hedgehogs, which are a common host of *H. longicornis* [50,51].

*Morganella* was the dominant genus in the deer group, and its relative abundance was the highest. *Morganella* is a gram-negative bacterium that is widely distributed in nature, and it colonizes mucous membranes of the gastrointestinal tract in humans and animals [52]. *Morganella* is currently are classified into *M*. *morganii* specie and *M*. *psychrotolerans* specie. *M. morganii* are classified into two subspecies, *Morganella morganii* and *M. sibonii* [53]. In this study, the relative abundance of *Morganella* in the deer group was higher than that in tigers, and the genus *Morganella* in both groups of samples was identified as *M. morganii*. *M. morganii* is considered to be an increasingly important pathogen due to its enhancing virulence and resistance to β-lactam antibiotics [54]. A fox had abundant ear discharge and secondary infections of *M*. *morganii*, which were suspected to be from ear mite parasites [55]. Klubal et al. (2016) detected *Morganella* in *Ixodes ricinus*, accounting for 3% [56]. These results indicate that *M. morganii* can colonize the midgut of *H. longicornis*.

In a similar study, *R. microplus* ticks were collected from cattle and goats in Jiangxi, Hunan, Guizhou and other provinces in China, and their intestinal microbes were analyzed [57]. *Rickettsia peacockii* and *Coxiella* spp. were the dominant strains of the *R. microplus* ticks from cattle, while *Uncultured bacterium* and *Pseudomonas* were the most abundant in all samples from goats [57]. Also, the bacterial diversity in the sample from Hunan was the highest among all tick samples, indicating that the diversity of tick intestinal microbes was affected by the types of tick hosts and geographical locations [57]. In addition, *R. microplus* was collected from two distinct ecological regions in Northern and Middle Magdalena, and the bacterial diversity of their salivary glands and intestines was studied by culture-dependent methods [58]. The results show that Proteobacteria were dominant in Northern Magdalena, while Firmicutes were the most abundant in Middle Magdalena, suggesting that the diversity of bacteria might be affected by geographical distribution [58].

The bacteria compositions of the *H. longicornis* parasitizing tiger and deer were shown to be different by high-throughput sequencing technology. However, this study did not take into account of the effects of host’s immune status, physiological habits, and the interactions between ticks and hosts on the structure of microbiomes. The host animals were found to be physiologically normal and had not been vaccinated against tick-borne diseases, but the other immune statuses of the hosts was unknown. Also, the present study only used a sequencing-based approach that may not capture the entire microbial diversity present in the tick sample. For instance, the fungal component of the tick microbiota was neglected; it plays an important role in tick ecology and may interact with bacteria to influence host–pathogen dynamics. Culture-based methods are further needed to provide a more comprehensive understanding of the tick microbiota.

## 5. Conclusions

The relative abundance of bacteria in the midgut microbiota structure of blood-saturated *H. longicornis* parasitized tiger and deer was significantly different at the phylum and genus levels. 

## Figures and Tables

**Table 1:** PCR primer sequences for identification of ticks and *Morganella*.

Primer Names	Primer Sequences (5′→3′)
16S rDNA-forward ^1^	5′-CTGCTCAATGATTTTTTAAATTGCTGTGG-3′
16S rDNA-reverse ^1^	5′-CCGGTCTGAACTCAGATCAAGT-3′
COX1-forward	5′-GGAACAATATATTTAATTTTTGG-3′
COX1-reverse	5′ATCTATCCCTACTGTAAATATATG-3′
ITS-forward	5′-CGAGACTTGGTGTGAATTGCA-3′
ITS-reverse	5′-TCCCATACACCACATTTCCCG-3′
16S rDNA-forward ^2^	5′-AGAGTTTGATCCTGGCTCAG-3′
16S rDNA-reverse ^2^	5′-GGTTACCTTGTTACGACTT-3′
dnaN-foward	5′-ATGAAATTTACCGTTGAACGTGA-3′
dnaN-reverse	5′-CCATCCACCAGCTTCGAGGT-3′
tuf-foward	5′-TTGTTGCTGCAACTGATGG-3′
tuf-reverse	5′-CACCTTCATCTTTGCTCAG-3′
hdc-foward	5′-TCHATYARYAACTGYGGTGACTGGRG-3′
hdc-reverse	5′-CCCACAKCATBARWGGDGTRTGRC-3′

^1^ Primer sequences were used to identify ticks. ^2^ Primer sequences were used to identify *Morganella*.

**Table 2:** Indices of microbial abundance and diversity in two groups.

Group	Chao1	Good’s Coverage	Shannon	Simpson
Deer host	31.000	1.000	1.526	0.573
Tiger host	138.000	1.000	2.319	0.647

## Data Availability

The raw tags were deposited in the Sequence Read Archive (SRA) of the NCBI under BioProject accession number PRJNA1010648. The individual run files received accession numbers SRR25820263 and SRR25820262.

## Supplementary Materials

The following supporting information can be downloaded at: https://www.mdpi.com/article/10.3390/ani14111557/s1, Figure S1: Electrophoretogram of the COX1 and 16S rDNA genes and ITS sequences from fully engorged female *Haemaphysalis longicornis* parasitizing the surface of tiger and deer. M. DNA marker. 1, 2. Products of the COX1 gene from the female *Haemaphysalis longicornis* parasitizing the surface of tiger. 3, 4. Products of the COX1 gene from the female *Haemaphysalis longicornis* parasitizing the surface of deer. 5, 6. Products of the ITS sequences from the female *Haemaphysalis longicornis* parasitizing the surface of tiger. 7, 8. Products of the ITS sequences from female *Haemaphysalis longicornis* parasitizing the surface of deer. 9, 10. Products of the 16S rDNA gene from the female *Haemaphysalis longicornis* parasitizing the surface of tiger. 11, 12. Products of the 16S rDNA gene from the female *Haemaphysalis longicornis* parasitizing the surface of deer. N1, N2, N3. Negative control; Table S1: The relative abundances of 10 common bacterial genera and all unique bacterial genera in the two samples.
