# Supplementary material for: Composition of the Midgut Microbiota Structure of Haemaphysalis longicornis Tick Parasitizing Tiger and Deer
Source: Animals (Basel). 2024 May 24;14(11):1557. doi: 10.3390/ani14111557 (PMC11171073; doi:10.3390/ani14111557)
Supplement: Supplementary file 1 [file animals-14-01557-s001.zip › Table S1.pdf]

Table S1 The relative abundance of all bacterial genera in two groups.

Bacterial genera	Relative abundance of bacterial genera in each sample		Bacterial genera	Relative abundance of bacterial genera in each sample	
	deer	tiger		deer	tiger
Corynebacterium	0.00022	0.00308	*Aggregatibacter*	0	0.00022
Enterorhabdus	0.00002	0.00242	*Eubacterium*_ruminantium_group	0	0.00019
Serratia	0.00019	0.00235	*Ruminococcus*_torques_group	0	0.00019
Gemella	0.00015	0.00184	*Enhydrobacter*	0	0.00019
Streptococcus	0.00019	0.00116	*Ornithinibacillus*	0	0.00017
Halomonas	0.00012	0.00068	Lachnospiraceae_NK4A136_group	0	0.00409
Pseudomonas	0.00029	0.00056	UCG-002	0	0.00017
Eubacterium_coprostanoligenes_group	0.00002	0.00048	*Achromobacter*	0	0.00017
Sphingobium	0.00002	0.00012	*Lawsonella*	0	0.00017
Rodentibacter	0.00046	0.00005	*Sporosarcina*	0	0.00017
Diplorickettsia	0.00244	0.45717	*Nocardioides*	0	0.00024
Coxiella	0.00889	0.37688	*Desulfovibrio*	0	0.00024
Morganella	0.97972	0.04091	*Chryseobacterium*	0	0.00024
Aerococcus	0.00300	0.02737	*Lachnoclostridium*	0	0.00024
Staphylococcus	0.00033	0.00763	*Brevibacterium*	0	0.00012
Jeotgalicoccus	0.00104	0.00668	*Candidatus*_Saccharimonas	0	0.00027
Ralstonia	0.00130	0.00629	NK4A214_group	0	0.00017
Acinetobacter	0.00026	0.00026	*Kurthia*	0	0.00017
Bacillus	0.00026	0.01187	*Neisseria*	0	0.00017
Lactobacillus	0.00055	0.00261	*Sphingomonas*	0	0.00015
Psychrobacter	0	0.00799	*Akkermansia*	0	0.00015
Muribaculaceae	0	0.00203	*Candidatus*_Arthromitus	0	0.00145
Gordonibacter	0	0.00092	*Gottschalkia*	0	0.00012
Blautia	0	0.00078	*Rubellimicrobium*	0	0.00012
Escherichia-Shigella	0	0.00075	*Paracoccus*	0	0.00012
*Clostridia*_UCG-014	0	0.00230	*Anaerofustis*	0	0.00012
Klebsiella	0	0.00170	*Jeotgalibacillus*	0	0.00012
Facklamia	0	0.00150	*Romboutsia*	0	0.00012
Faecalibacterium	0	0.00048	*Chloroplast*	0	0.00012
Lactococcus	0	0.00048	*Marvinbryantia*	0	0.00012
Sutterella	0	0.00046	*Methylobacterium-Methylorubrum*	0	0.00012
Nitriliruptor	0	0.00036	*Brachybacterium*	0	0.00012
Roseburia	0	0.00036	*Nesterenkonia*	0	0.00012
Dietzia	0	0.00031	*Agathobacter*	0	0.00029
Adlercreutzia	0	0.00031	*Parasutterella*	0	0.00029
Lachnospira	0	0.00027	*Atopostipes*	0.00036	0
Skermanella	0	0.00012			
