# Supplementary figures and images for: Composition of the Midgut Microbiota Structure of Haemaphysalis longicornis Tick Parasitizing Tiger and Deer
Source: Animals (Basel). 2024 May 24;14(11):1557. doi: 10.3390/ani14111557 (PMC11171073; doi:10.3390/ani14111557)

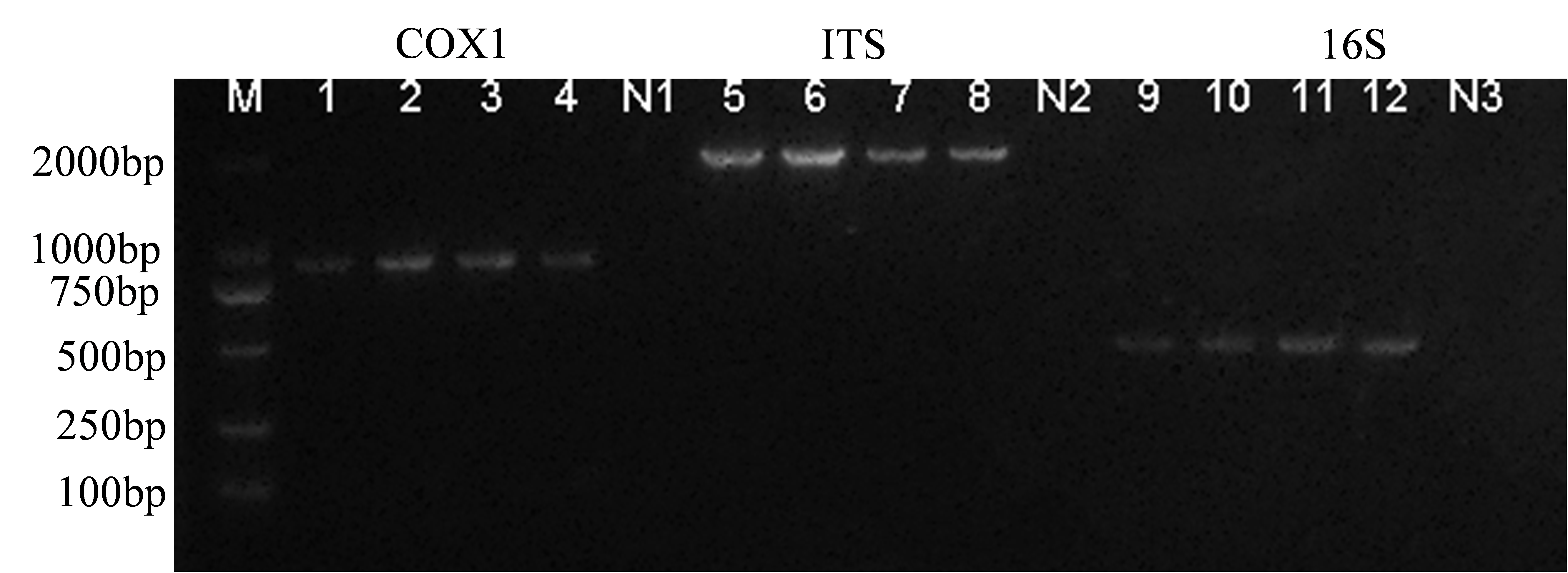

Supplement: Supplementary file 1 [file animals-14-01557-s001.zip › Figure S1.tif]
